# Monocaprin eye drop formulation to combat antibiotic resistant gonococcal blindness

**DOI:** 10.1038/s41598-020-68722-8

**Published:** 2020-07-21

**Authors:** Colin P. Churchward, Ali A. Al-Kinani, Hamdy Abdelkader, Julian Swinden, Opeoluwa Siwoku, Thinuba Varnakulasingam, Raid G. Alany, Lori A. S. Snyder

**Affiliations:** 1 School of Life Sciences, Pharmacy, and Chemistry, Kingston University, Penrhyn Road, Kingston upon Thames, UK; 2 Pharmaceutics Department, Faculty of Pharmacy, Minia University, Minia, Egypt; 3 Present Address: National Heart and Lung Institute, Imperial College London, Guy Scadding Building, Royal Brompton Campus, London, SW3 6LY UK

**Keywords:** Bacteriology, Antimicrobials, Drug development

## Abstract

*Neisseria gonorrhoeae* bacteria are acknowledged as an urgent threat to human health because this species has developed resistances to all of the antibiotics used clinically to treat its infections. *N. gonorrhoeae* causes the sexually transmitted disease gonorrhoea, but also causes blindness when the bacteria infect the eyes. Infants are particularly susceptible, acquiring the infection from their mothers at birth. We have shown that the monoglyceride monocaprin rapidly kills *N. gonorrhoeae* and other bacterial species and is non-irritating in ocular assays. Here we show that the physical and chemical properties of monocaprin make it ideal for use in a thickened eye drop formulation to combat eye infections. Monocaprin-containing formulations were assessed using analytical techniques and for antimicrobial activity in vitro and in ex vivo infections. Monocaprin-containing formulations retained activity after three years and are non-irritating, unlike preparations of povidone iodine in our assays. A recommended formulation for further development and investigation is 0.25% monocaprin in 1% HPMC with 1% polysorbate 20.

## Introduction

Once a drug to combat infection has been discovered, then formulation is required so that the drug can effectively be converted to a form that could be used to clear a particular infection from a patient. Monocaprin has been proposed as a treatment and prophylaxis for *Neisseria gonorrhoeae* and other bacterial eye infections^1–3^. *N. gonorrhoeae* is a bacterial species that causes the sexually transmitted infection gonorrhoea and as a result can be transferred to the eyes of infants during childbirth causing ophthalmia neonatorum^4^. Autoinnoculation can also result in adult eye infections^5^. Progressive development of resistance to all antibiotics used against *N. gonorrhoeae* clinically^6^ presents a problem for prophylaxis and treatment of ophthalmia neonatorum and treatment of adult gonococcal eye infections. Prophylaxis for ophthalmia neonatorum has involved the use of substances applied within an hour of birth that have prevented the establishment of gonococcal infection^4,7–8^. Silver nitrate was the first prophylaxis and significantly reduced cases of gonococcal blindness, however it has been discontinued in most areas due to issues with chemical conjunctivitis and toxicity^7^. Some cases of ophthalmia neonatorum still developed with silver nitrate, which is unable to treat an active infection^7^. Antibiotics erythromycin and tetracycline, in ocular ointments, have largely replaced silver nitrate as prophylaxis, however resistance to these antibiotics emerged in *N. gonorrhoeae* in the 1980’s^9^. Although povidone iodine has been suggested for prophylaxis it is not currently recommended by the CDC^10^ and has similar issues as silver nitrate, both in terms of ocular irritation and inability to treat active infections^7–8,11^. The World Health Organisation recommendations for treatment of gonococcal eye infections are: 50 mg/kg ceftriaxone; 25 mg/kg kanamycin; or 25 mg/kg spectinomycin, each as a single IM dose, noting that “the choice of treatment may depend on the cost and quality of the medicines in different settings and on equality considerations^12^.” The 2019, British Association for Sexual Health and HIV national guidelines for the management of infection with *N. gonorrhoeae* recommend treatment of gonococcal conjunctivitis with 1 g ceftriaxone intramuscularly as a single dose^13^. This advice is based on successful treatment of adults in 1989^14^ and there is no mention of an alternative suggestion in cases of ceftriaxone resistant infections. Given cases of ceftriaxone resistant *N. gonorrhoeae* and wide-spread multi-drug resistance^6^, an alternative is needed to prevent blindness from these bacteria. In 1880, before the advent of antibiotics, up to 79% of children in institutions for the blind had suffered from gonococcal ophthalmia neonatorum at birth^15^. The infection can progress rapidly, with the bacteria perforating the globe of the eye and causing blindness within 24 h^4–5,15^.

We have identified that monocaprin is able to kill *N. gonorrhoeae*, including clinical isolates of *N. gonorrhoeae*, the related species *Neisseria meningitidis*, which can also cause eye infections, and other keratitis causing bacteria, including *Chlamydia trachomatis*, another major cause of ophthalmia neonatorum^1,3,16^. Monocaprin has been shown to retain its antimicrobial activity in artificial tear fluid, is devoid of corneal and conjunctival irritation, kills all bacteria present within 2 min, and *N. gonorrhoeae* have been unable to develop resistance to monocaprin^1–2^. A monocaprin-containing prophylaxis would prevent the establishment of infection, while a treatment would clear an established infection. The rapid bactericidal activity of monocaprin indicates a single formulation is likely to achieve both outcomes.

## Results and discussion

### Considerations for a monocaprin-based ocular formulation

To be effective, a formulation against ocular infections must distribute to the corneal surface, conjunctiva, and tear drainage ducts and remain there long enough to exert the antibacterial activity. In seeking an antimicrobial agent, killing activity of 2 min was selected rather than a longer time period, such as 1–24 h, because this is a realistic exposure time achievable on the eye^1^.

The hydrophilic-lipophilic balance (HLB) value for monocaprin is 7.5, calculated via the Davies’ method^17^. It is a monoglyceride that is a waxy solid powder at room temperature, with a melting temperature of 51–55 °C, which is poorly water soluble, but highly soluble in ethanol. Direct analytic detection of monocaprin for development of pharmaceutical products has previously used high performance liquid chromatography (HPLC) based mass spectrometry detection to identify monoglycerides down to 1–30 ppm^18^.

### Eye ointment formulations

Currently in use are eye ointments as prophylaxis, containing erythromycin or tetracycline^10,12^. Such semi-solid dosage forms are retained on the eye for longer periods than eye drops, therefore improving ocular drug levels^19^. Lack of aqueous solubility of monocaprin is not an issue for this type of formulation, as the ointment base, white petrolatum USP, is non-aqueous.

**Figure 1:**
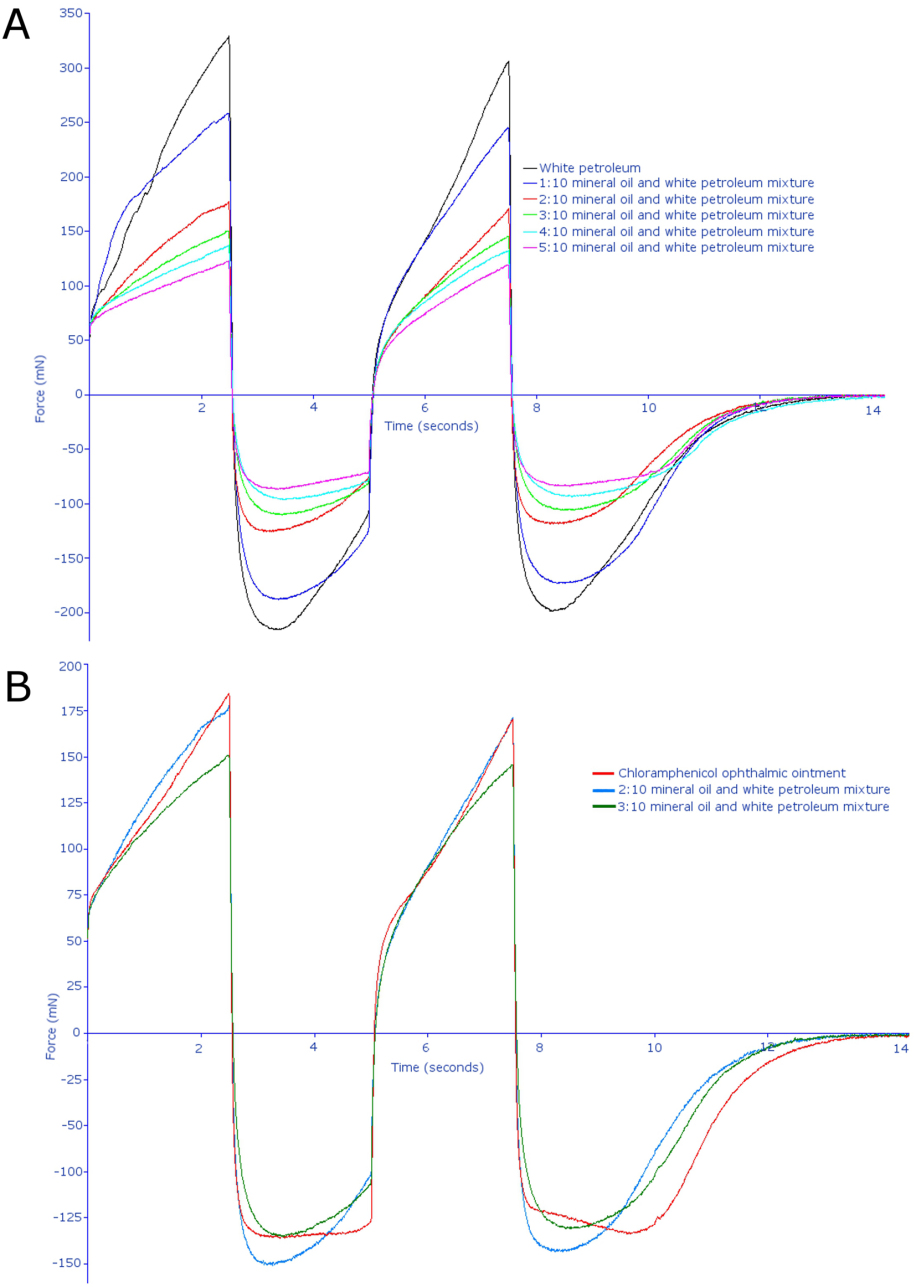
Texture analysis of ointments. (**A**) Comparison of the texture of different blends of light mineral oil and white petroleum at ratios of 1:10 (blue), 2:10 (red), 3:10 (green), 4:10 (light blue), and 5:10 (pink), and white petroleum alone (black). (**B**) Comparison of chloramphenicol ophthalmic ointment to the blends of light mineral oil and white petroleum, showing that chloramphenicol ophthalmic ointment (red) most closely resembles a 2:10 light mineral oil: white petroleum blend profile (blue). Graphs in both panels represent a mean of five sets of analysis on each sample by the Stable Microsystems TA.XTplus Texture Analyser and were generated by the Stable Microsystems Exponent Lite software v6.1.4.0.

The mechanical properties of commercially available 1% (w/w) chloramphenicol ophthalmic ointment was investigated and compared with different blends of light mineral oil and white petroleum (Fig. 1A). This data indicated that the mechanical properties of the commercial ophthalmic ointment can be replicated with a blend of two parts light mineral oil to ten parts white petroleum. The initial measure of parts for light mineral oil was by volume and the measure of parts for white petroleum was by weight. As light mineral oil has a density of 0.84 g/cm^3^, this means that this mixture is equivalent to approximately 15% by weight light mineral oil and 85% by weight white petroleum.

The texture analysis graph for chloramphenicol ointment (Fig. 1B) and the blends of the light mineral oil and white petroleum match in terms of the mechanical properties of hardness (positive peak 1 height), cohesiveness (positive peak 1 area), and adhesiveness (negative peak 1 area; Fig. 1B) are presented. Monocaprin concentrations of 0.062%, 0.125%, 0.188%, and 0.25% by weight were incorporated in the developed ointment base with no sign of phase separation or precipitation upon storage.

### Eye drop formulations

Eye drops are sterile liquid dosage forms, which may be unsuitable carriers for monocaprin given its poor solubility in water. However, solubility can be improved through the addition of excipients^20^. In addition, viscosity enhancers can be added to eye drops to prolong the time they are retained on the eye, such as carboxymethylcellulose (CMC, E number E466) and (Hydroxypropyl)methyl cellulose (HPMC, E number E464), both long chain polymers derived from cellulose and commonly used in food and pharmaceutical products^21^. Ophthalmic viscosity enhancing polymers CMC and HPMC are both widely used in ophthalmic preparations and have good safety records^19–20^.

**Figure 2:**
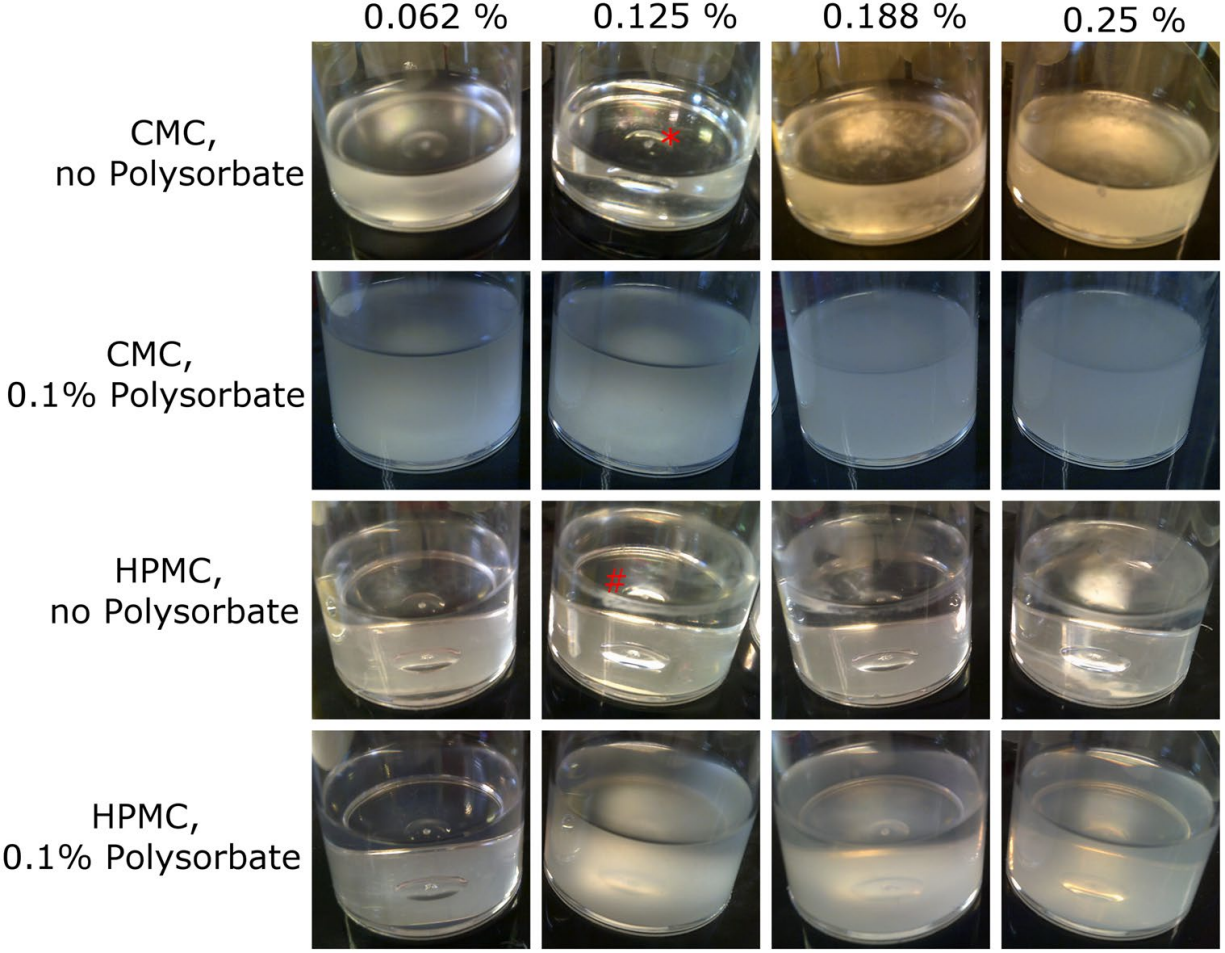
Comparison of the influence of polymer and polysorbate 20 on the solubility of monocaprin in thickened eye drop formulations. In CMC-based formulations, monocaprin forms clumps (indicated by a red asterisk) at 0.125% and above. Addition of 0.1% polysorbate 20 evenly distributed the monocaprin, although it did not fully solubilise. In HPMC-based formulations, monocaprin forms a fine precipitate just under the meniscus at 0.125% and above (indicated by a red hash). Addition of 0.1% polysorbate 20 solubilised the monocaprin.

Monocaprin concentrations in the liquid dosage formulations were 0.062%, 0.125%, 0.188%, and 0.25%, comparable to the ointment. To enhance solubility of monocaprin, it was first dissolved in propane-1,2-diol, which would improve the aqueous solubility of the monocaprin and would be less likely to cause ocular irritation than other solvents. The monocaprin was then combined with the viscosity enhancing polymer. However, monocaprin precipitated in CMC-based formulations and a cloudy layer formed at the top of the vessel just below the meniscus in HPMC-based formulations (Fig. 2). The addition of 0.1% (v/v) polysorbate 20 (Tween 20) improved monocaprin solubility (Fig. 2). At this concentration of polysorbate 20, the monocaprin was completely in solution in the HPMC polymer-based formulations, but this was not the case in the CMC-based formulations (Fig. 2). This indicates that HPMC is the polymer of choice for further formulation development.

### Antimicrobial activity of formulations

**Figure 3:**
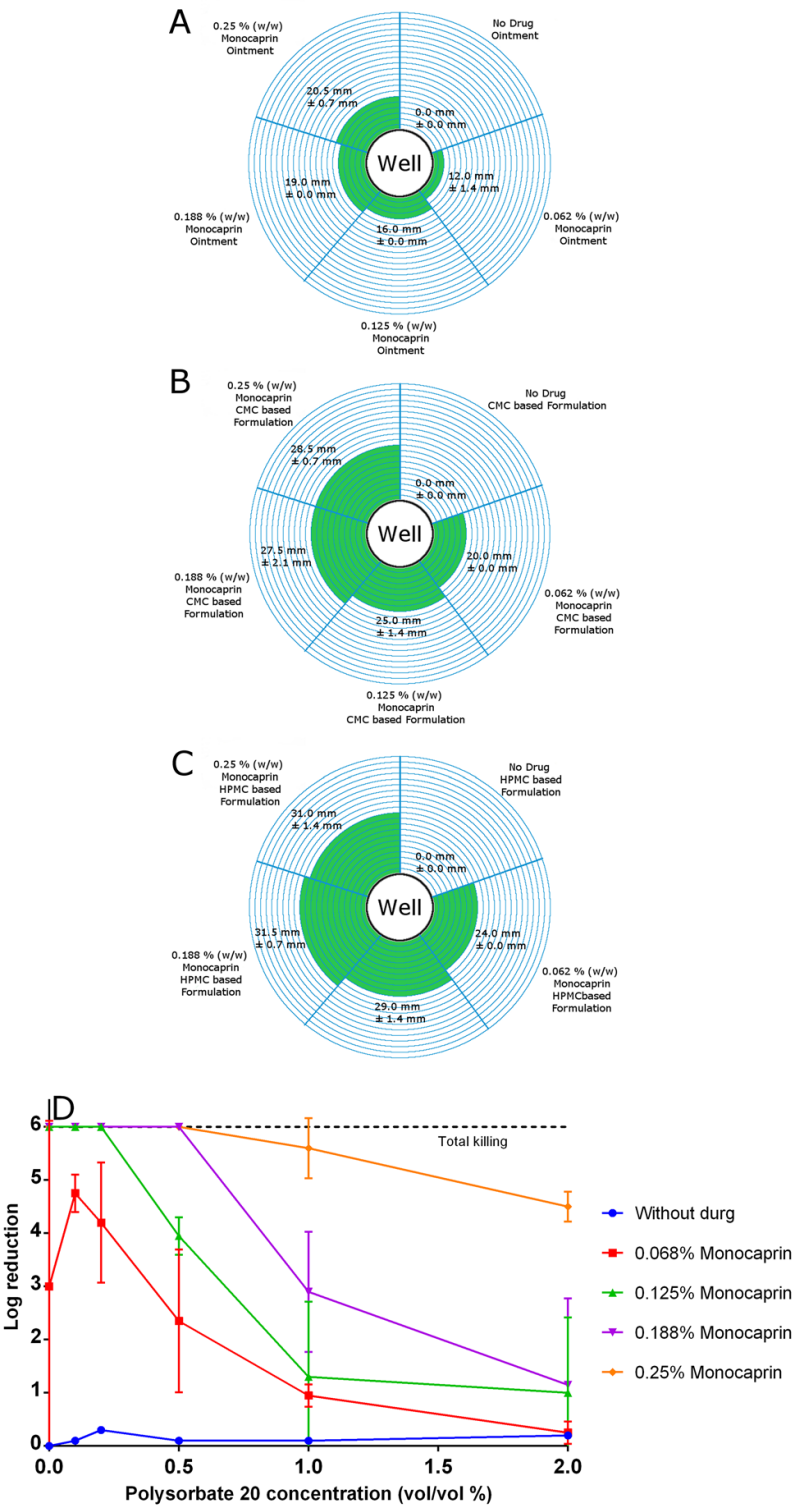
Bacteriostatic and bactericidal activity of monocaprin-containing formulations. (**A**–**C**) Growth inhibition zones of all formulations. Sizes of zones of inhibited *N. gonorrhoeae* strain NCCP11945 growth represented in 2 mm rings of green around an 8 mm well with actual sizes in mm, as indicated. (**A**) Ointment formulations with 0.062%, 0.125%, 0.188%, and 0.25% monocaprin and a no monocaprin control. (**B**) CMC-based formulations with 0.062%, 0.125%, 0.188%, and 0.25% monocaprin and a no monocaprin control. (**C**) HPMC-based formulations with 0.062%, 0.125%, 0.188%, and 0.25% monocaprin and a no monocaprin control. (**D**) Log reduction of viable *N. gonorrhoeae* exposed for 2 min to HPMC-based formulations containing 0%, 0.062%, 0.125%, 0.188%, and 0.25% monocaprin and 0%, 0.1%, 0.2%, 0.5%, 1%, and 2% polysorbate 20. Values over 4 log_10_ are bactericidal (blue dashed line); those at 6 achieved total killing of all gonococci in the experiment (black dashed line).

Thickened eye drop and ointment dosage formulations were assayed for bacteriostatic activity and bactericidal activity via bacterial growth inhibition assays and bacterial log reduction assays, respectively. Ointments produced the smallest zones of inhibition at 12.0 mm for 0.062% to 20.5 mm for 0.25% monocaprin, likely due to a slower diffusion rate of the monocaprin from the well into the agar compared to the liquid dosage formulations (Fig. 3A). The HPMC-based formulations, producing zones of inhibition of 24.0 mm for 0.062% to 31.0 mm for 0.25% monocaprin (Fig. 3C), performed slightly better than CMC-based formulations, with zones of 20.0 mm for 0.062% to 28.5 mm for 0.25% monocaprin (Fig. 3B). It was revealed that polysorbate 20, included in the eye drop formulations, had some antimicrobial activity against *N. gonorrhoeae* on its own. The mean zone of inhibition with polysorbate 20 alone was 13 mm, whereas the zones of inhibition for the CMC and HPMC-based monocaprin formulations started at 20 and 24 mm, respectively (Fig. 3B, C).

Based on producing larger zones of inhibition (Fig. 3C) and being the polymer of choice for solubility (Fig. 2), the HPMC-based thickened eye drops were assessed in log reduction assays, demonstrating that they are effective at killing *N. gonorrhoeae*, with over 4 log_10_ activity, the threshold needed for bactericidal activity (Fig. 3D). To evaluate if the observed antimicrobial activity of polysorbate 20 was bactericidal it was assessed alone at concentrations of 0.1%, 0.2%, 0.5%, 1%, 2%, and 3%. Even at the highest concentration tested, no bacterial killing was observed, showing that the action of the surfactant in the formulation is bacteriostatic. Surprisingly, however, the higher the concentration of polysorbate 20 in the formulations the lower the anti-gonococcal properties of the monocaprin (Fig. 3D). A similar effect has been reported in a previous study investigating polysorbates and antimicrobials^22^. This inhibition of the monocaprin by the surfactant appears to be concentration dependant; the greater the monocaprin concentration in the formulations the more polysorbate 20 is required to inhibit its action. To achieve a log_10_ reduction of 4 or greater, a maximum concentration of 0.2% polysorbate 20 should be used for 0.062% and 0.125% monocaprin formulations, 0.5% polysorbate 20 for 0.188% monocaprin formulations, and 1% polysorbate 20 for 0.25% monocaprin formulations. It is plausible that polysorbate 20 and monocaprin form mixed micelles, decreasing the availability of the free monocaprin and consequently reducing the anti-gonococcal properties of the formulation.

Both the semi-solid and liquid dosage forms had strong bacteriostatic activity, however only the liquid dosage forms were bactericidal. This could mean that the ointment, when applied to the eye, may be able to stop the growth of gonococci but may not kill gonococcal cells in the tear fluid. This may be sufficient for a prophylaxis, but not for a treatment. It appears that release of the monocaprin from the ointment base is insufficient to reach a level where it would actively kill the bacterial cells. In comparison, the thickened eye drop formulation killed all of the gonococcal cells present in the simulated tear fluid, therefore making it a preferred candidate for future clinical studies because it can be used for both prophylaxis to prevent establishment of infection and treatment of active infections.

**Figure 4:**
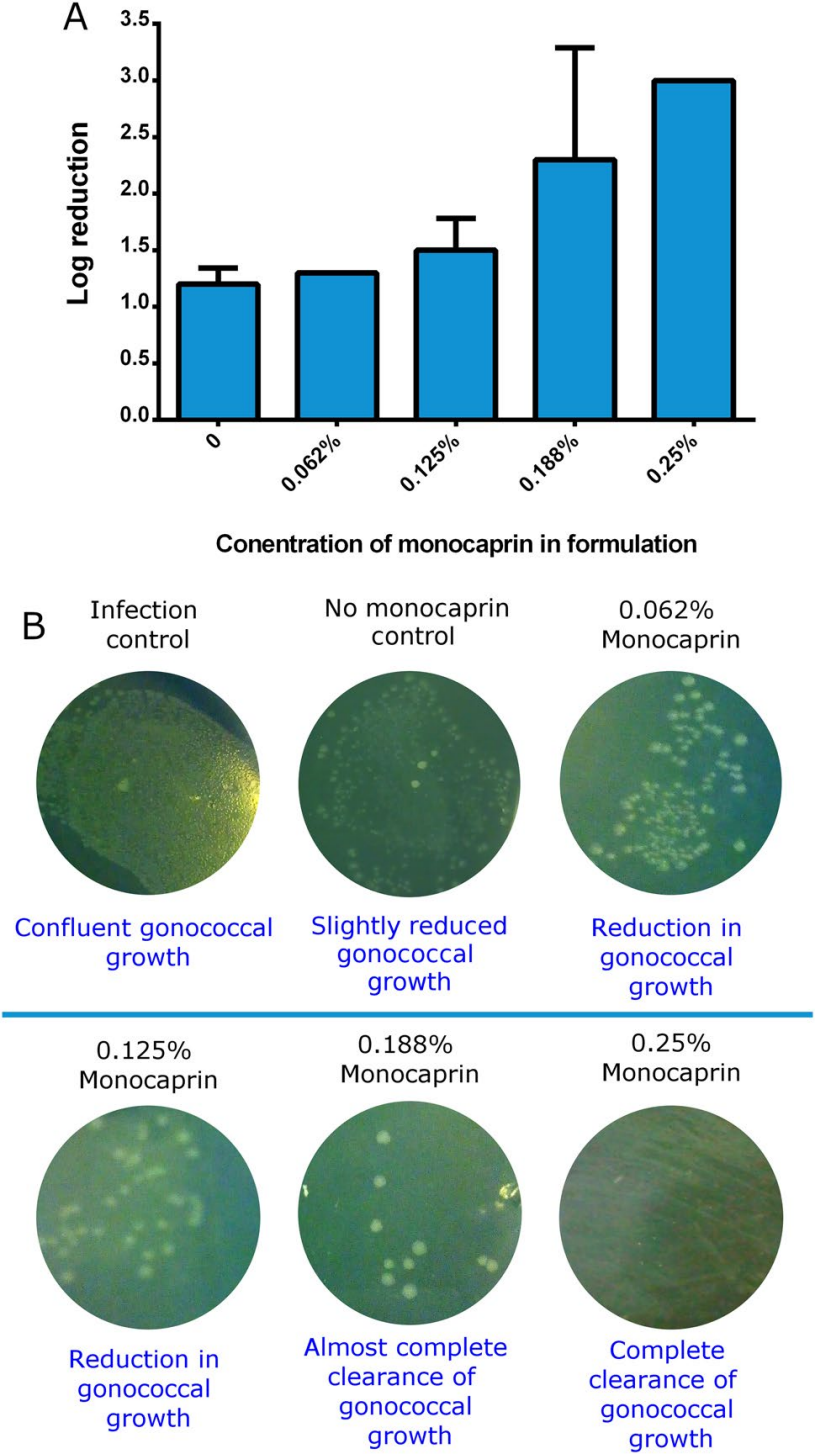
Clearance of *N. gonorrhoeae* from the surface of eyes using an HPMC-based monocaprin-containing ophthalmic formulation. (**A**) Log reduction in the viable *N. gonorrhoeae* recovered from an explanted corneal infection model treated with increasing concentrations of monocaprin in an HPMC-based formulation. Due to statistical limits of the assay, the maximum reduction possible was 3 log_10_ at which point total killing of all *N. gonorrhoeae* was achieved. Error bars represent standard deviation over two experiments. Due to identical results between experiments, the error bar for 0.062% is not visible. There was complete killing of *N. gonorrhoeae* in both 0.25% monocaprin experiments, thus no error bars are shown as the maximum value was achieved in both cases. (**B**) Explanted corneal infection model assessment via printing of the corneal surface onto a GC agar plate, revealing viable *N. gonorrhoeae* growth, as indicated. Confluent growth of *N. gonorrhoeae* is observed on untreated corneas, with corresponding reduction in gonococcal growth with increasing monocaprin concentrations. Complete clearance is achieved at 0.25% monocaprin.

The thickened eye drop and ointment formulations were assessed using an ex vivo corneal infection model to evaluate the viability of *N. gonorrhoeae* on the surface of an eye following treatment. Excised bovine corneas were maintained in MEM and infected with approximately 10^6^ gonococcal cells. Saponin was used to lyse the cells of the epithelial layer of the corneas to release attached and internalised gonococci. A reduction in bacteria recovered from the corneas (Fig. 4A) and in bacteria printed onto GC agar from the corneal surface (Fig. 4B) was seen in all corneas treated within an hour of infection, as would be the case for prophylaxis. Reduction in gonococci is also seen with the application of a 0% monocaprin, 0.5% polysorbate 20 HPMC-based formulation (Fig. 4), which suggests that the surfactant is contributing to reduction in bacteria. In addition to the antimicrobial activity observed, the surfactant may also interfere with the adhesion of the gonococci to the epithelial layer. Therefore, inclusion of polysorbate 20 in the formulation is beneficial both to improve the solubility of the monocaprin and also to contribute to the prevention of gonococcal infection. Using this model system, it has been possible to demonstrate complete clearance of infection of *N. gonorrhoeae* from the eye (Fig. 4).

### Monocaprin antimicrobial activity and detection following storage.

Following nine months of storage at room temperature in the dark, the HPMC- and CMC-thickened eye drop formulations were still as antimicrobially effective as newly made formulations, assessed by growth inhibition assays, as previously. To assess the stability of the monocaprin in the formulations after nine months, the published HPLC method for monocaprin detection was used^18^, with a minor modification. As the monocaprin peaks were wide and gave poor resolution at lower concentrations, a longer 250 mm column based on solid core particles (5 µm in diameter) was used, which increased the resolution. The final retention time of monocaprin using this modified method was approximately 3.4 min. The test performed well in regression analysis with a R^2^ value of 0.9997, with good inter-day and intra-day repeatability, precision, and accuracy. The International Council for Harmonisation of Technical Requirements for Registration of Pharmaceuticals for Human Use (ICH) guidelines only give a brief outline of how to set the limits of detection (LoD) and limits of quantification (LoQ) as there are a few methods that can be used^23^. The LoD and LoQ here were determined to be 13.4 and 40.6 µg/ml, respectively. Using this method, the formulations stored for nine months had no detectable decrease in monocaprin nor the presence of any degradation products. At 35 months after preparation of the formulations, antimicrobial activity was still evident via growth inhibition assays.

### Comparison with povidone iodine

**Figure 5:**
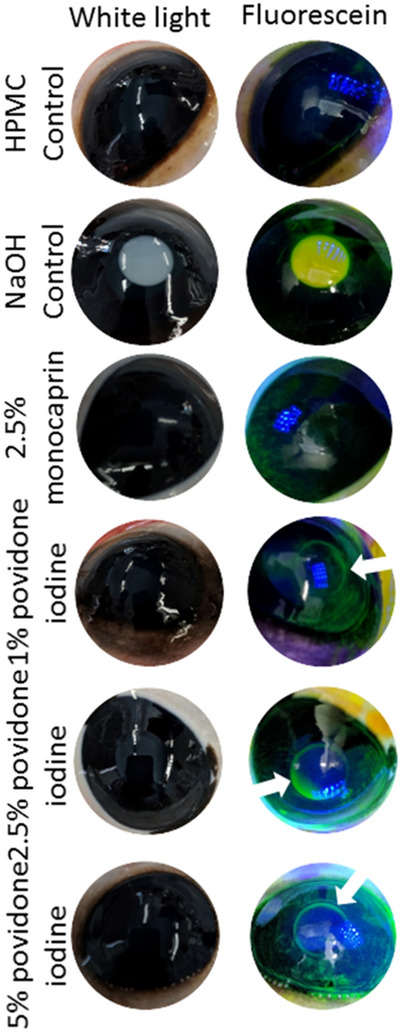
Bovine corneal opacity and permeability (BCOP) assay reveals povidone iodine irritation makes monocaprin more suitable for prophylaxis. Monocaprin has previously been shown to be non-irritating in several assays^1^. Povidone iodine, however, is known to cause irritation^7,8,11^. Comparison with the sodium hydroxide control, a strong ocular irritant, the BCOP assay indicates povidone iodine is a slight ocular irritant, according to the scoring scheme^30^, where opacity is observed (white arrows in fluorescein staining), yet it is not confluent. This irritation increases with concentration (bottom three rows). Here a concentration of monocaprin 10 × higher than proposed in the eye drop formulation is shown to be non-irritating (2.5% monocaprin) and indistinguishable from the negative control (top row). Images on the left are under white light and those on the right are with a cobalt light filter following fluorescein staining to highlight corneal damage to the area of application of the substance to the surface of the eye.

Comparison of these formulations with povidone iodine, which has been proposed as an alternative prophylaxis for ophthalmia neonatorum, demonstrates that the monocaprin-based thickened eye drops are superior. The recommended concentrations of povidone iodine for prophylaxis^11^ resulted in signs of irritation using the bovine corneal opacity and permeability (BCOP) assay, whereas the monocaprin formulation did not (Fig. 5). Credé’s silver nitrate prophylaxis has been discontinued and is unavailable in much of the world due to its reported ocular irritation and resulting chemical conjunctivitis, as well as treatment failures^7^. Cases of toxic chemical conjunctivitis and treatment failures are reported with povidone iodine^7,8,11^, consistent with the irritation results seen here (Fig. 5). These results suggest that monocaprin-containing formulations are a superior choice and a promising alternative for ophthalmia neonatorum prophylaxis.

Although a monocaprin ophthalmic formulation still needs to be assessed in humans, it is envisaged that for treatment, patient eyes would be rinsed or wiped with normal saline to clear excess discharge and non-adherent bacteria, then the monocaprin eye drop would be instilled. It is not known if more than one application will be necessary, however complete killing of *N. gonorrhoeae* can be achieved at 0.188% and 0.25% monocaprin in our models (Figs. 3D, 4). As with other forms of prophylaxis, it is envisaged that the monocaprin formulation would be applied within an hour of birth or as soon as possible after accidental autoinoculation in the case of adult exposures. A single dose application should be sufficient for prophylaxis due to the relatively lower bacterial load and extent of dissemination to ocular tissues versus treatment of an active infection. We propose single-use applicator packaging for the purposes of infection control. The eye drop formulations are relatively easy to make, from inexpensive components, which could be extemporaneously prepared in areas of urgent need where antimicrobial resistant *N. gonorrhoeae* incidence rates are high. During formulation investigations, it was found that at the final concentrations determined to be best for the HPMC-based thickened eye drop, all components combined readily. Given the activity of monocaprin against *C. trachomatis*^16^, *N. meningitidis*, and other bacteria capable of causing ocular infections^3^, this formulation has strong potential to replace current prophylaxis and treatments.

In addition to its antibacterial activity, monocaprin also has known antiviral activity against enveloped viruses, including herpes simplex virus type 2 (HSV-2), human immunodeficiency virus type 1 (HIV-1), vesicular stomatitis virus, visna virus, respiratory syncytial virus (RSV), and parainfluenza virus type 2, which are all enveloped viruses like SARS-Cov-2^16,22,24–26^. Indeed, it has been proposed that antiviral fatty acids may have a role in aiding to decrease morbidity and mortality in COVID-19^26^. Therefore, there may be use for an ocular formulation such as this, for example to supplement PPE for health care workers in high-risk areas, where use during and/or at the end of shifts working with COVID-19 positive patients may reduce transmission or reduce the initial viral load.

In conclusion, a thickened eye drop formulation of monocaprin in HPMC with polysorbate 20 has been demonstrated to have rapid and complete killing activity of *N. gonorrhoeae *in vitro and ex vivo, is biocompatible according to ocular irritation assays, and is stable and retains its antimicrobial activity. The recommended formulation comprise 0.25% monocaprin in 1% HPMC with 1% polysorbate 20. At these concentrations all components dissolve effectively in sterile distilled water forming a simple aqueous solution, ready to use eye drop.

## Methods

### Texture analysis

Blends of light mineral oil and white petroleum were made by adding one, two, three, four, or five parts light mineral oil (0.84 g/mL at 25 °C; Sigma-Aldrich) to 10 parts white petroleum blends (Vaseline; Sigma-Aldrich). The light mineral oil was mixed into the white petroleum on a black pill tile and sheered together using two 30 cm spatulas until an even consistency was obtained. To compare these to commercially available ophthalmic ointments, 1% (w/w) chloramphenicol ophthalmic ointment (GoldenEye, Typharm Limited) was used. Texture analysis of semi-solid samples used 10 ml wide-necked glass vials placed on a Stable Microsystems TA.XTplus Texture Analyser testing stage and clamped into position. The probe tested the sample twice, automatically, before the sample was removed, the surface flattened, and tested again. Each sample was thus tested five times. Stable Microsystems Exponent Lite (version 6.1.4.0) software was used to obtain data and to combine each of the five replicates into representative plots.

### Preparation of ophthalmic ointments

Monocaprin was warmed at 55 °C until melted, mixed with light mineral oil warmed to 37 °C, and added to the required amount of white petroleum on a black pill tile. The mineral oil was mixed and then sheered into the white petroleum using two 30 cm spatulas until an even consistency was obtained and the drug was evenly distributed throughout the ointment. Ointments were stored in 60 ml plastic pots (Sterilin Ltd) at room temperature until required.

### Preparation of CMC polymer-based thickened eye drops

A monocaprin stock of 100 g/L in propan-1,2-diol (BDH Chemicals, VWR International) was made. The required amount of carboxymethylcellulose (CMC) sodium salt (medium viscosity, 400–800 cP, 2% in H_2_O at 25 °C, Sigma-Aldrich) was added to half the required final volume of sterile deionised water (i.e. for 100 g formulation, 50 ml of water is added) in a sterile conical flask and placed on an orbital shaker at 200 rpm overnight at room temperature. A double concentrate of polysorbate 20 (BDH Chemicals, VWR International) was made up in sterile deionised water. The monocaprin stock was then added at double the required overall concentration and extra propan-1,2-diol was added (if required) to bring the concentration up to 10%. After briefly mixing this solution, the double concentrate of the polymer was added to the double concentrate of polysorbate 20, monocaprin, and propan-1,2-diol to the required weight. This thickened solution was mixed at room temperature on a Labnet Mini LabRoller (Edison, NJ, United States) with a 5 × 50 ml tube rotisserie.

### Preparation of HPMC polymer-based thickened eye drops

(Hydroxypropyl)methyl cellulose (HPMC) with an average polymer molecular weight of 90,000 (15,000 cP, 2 wt% in H_2_O at 20 °C, Sigma-Aldrich) was included in formulations using a similar method of preparation as CMC-based formulations. To produce the polymer gel, the required about of HPMC powder was added to the required amount of sterile deionised water heated to 90 °C. The double concentrate polymer was shaken on an orbital shaker at room temperature at 200 rpm until cooled. The polymer was then placed at 4 °C for an hour and then added to the double concentrate of polysorbate 20, monocaprin, and propan-1,2-diol as for the CMC-based formulations.

### Assessing bacteriostatic properties of formulations via growth inhibition tests

*Neisseria gonorrhoeae* strain NCCP11945 was cultured as previously^1^ overnight on a GC plates (Oxoid) with Kellogg’s supplements^27^ at 37 °C, 5% CO_2_. A cell suspension (0.5 McFarland standard) was streaked onto GC plates and a sterilised number 5 cork borer was used to make a well in the agar with a diameter of 8 mm. Approximately 150 µl of the test formulation was placed in the well. For testing of ointments, the semi-solid material was used to fill the well until full. Plates were incubated at 37 °C, 5% CO_2_ for 16–18 h. The diameters of the inhibition zones were measured.

### Assessing bactericidal properties of formulations via log reduction

Log reduction assays were conducted as previously^1^ with slight modifications; 250 µl of a double concentration of bacteria suspension (2 × 10^7^) was added to 0.25 g of the test formulation. All HPMC-based formulations were tested for two minutes, as previously^1^. Formulations without monocaprin were used as negative controls.

### Ex vivo infection of corneas

Assessment of clearance of bacteria from the corneal surface used corneas prepared similarly to those used for herpes simplex virus infection studies^28–29^. Excised bovine corneas (ABP, Guildford, UK) supported by scaffold medium of 0.2 g agarose in Minimum Essential Media (MEM, Gibco), were used for infection studies within 24 h. Corneal surfaces were infected with 100 µl of *N. gonorrhoeae* strain NCCP11945 at approximately 10^7^ cfu/ml and incubated for 1 h at 37 °C, 5% CO_2_. Corneas were then washed five times with sterile phosphate-buffered saline to remove non-adherent bacterial cells before application of 500 µl of the thicken eye-drop formulations containing 1.25% HPMC, plus varying concentrations of monocaprin and polysorbate 20: 0% monocaprin and 0.5% polysorbate 20 (no drug control); 0.062% monocaprin and 0.2% polysorbate 20; 0.125% monocaprin and 0.2% polysorbate 20; 0.188% monocaprin and 0.5% polysorbate 20; and 0.250% monocaprin and 0.5% polysorbate 20. After 2 min, formulations were washed off the corneas by dipping them in sterile PBS ten times.

Colony forming unit counts of surviving gonococcal cells were determined by incubating corneas in 1% saponin at 37 °C for 10 min. A clean scalpel blade was passed over the epithelial layer and then the saponin was used to wash the epithelial layer of any loose cells. Saponin was recovered, serially diluted in GC broth, and plated as for the log reduction assays. Plates were incubated at 37 °C, 5% CO_2_ for 48 h before counting. Additionally, treated and washed corneas were pressed against a GC plate for 30 s. Plates were incubated at 37 °C, 5% CO_2_ for 48 h before assessing them for bacterial growth.

### High performance liquid chromatography monocaprin detection

The HPLC-based monocaprin detection method was developed based on a previous method^18^ using a Perkin Elmer 200 series HPLC system with a series 225 auto-sampler and series 200EP diode array detector (UV/VIS). Data was acquired using Perkin Elmer TotalChrom Workstation (version 6.3.1). A 4.6 × 250 mm C18 column packed with 5 µm Kinetex solid core particles (Phenomenex) was used to analyse samples. An isocratic mobile phase of 80:20 (v/v) of acetonitrile/water was used at a flow rate of 1 ml/min. Samples were injected at a volume of 50 µl. Monocaprin was detected by UV absorption at 208 nm. A stock of 2 mg/ml monocaprin was made in acetonitrile. This was diluted in acetonitrile to monocaprin concentrations of 1,000, 750, 500, 250, 125, and 62.5 µg/ml. Five injections of each of these standards was made on the optimised method followed by five injections of acetonitrile (blank). For the intra-day and inter-day testing, monocaprin concentrations of 750, 250, 125, and 62.5 µg/ml were tested. Three injections of a 1,000 µg/ml monocaprin (standard) followed by a blank and then a sequence of the test samples with each being tested in triplicate and a standard and blank being run after every six injections. The limits of detection (LoD) and quantification (LoQ) were determined by a signal-to-noise approach. A signal-to-noise ratio of 3:1 was used to determine the LoD and 10:1 used for LoQ^23^.

### Bovine corneal opacity and permeability (BCOP) assay

The BCOP assay was performed as previously^1^. Bovine eyes (ABP, Guildford, UK) without visible damage were placed in weigh boats in a water bath at 37 °C for 10 min. An 8 mm rubber O-ring was placed on the centre of the cornea into which was added one drop of sterile saline before returning to the 37 °C water bath for five minutes. The saline was carefully removed and replaced with 100 µl of test substance, left of the eye for 30 s before washing with 10 ml of sterile saline and incubating for 10 min in the 37 °C water bath. After sodium fluorescein treatment (2% (w/v) pH 7.4), the cornea was examined under a cobalt blue filter (465–490 nm, peak 480 nm) and the eyes were scored for opacity^30^. Mean opacity was given a score between 0 and 4 (0, no opacity; 1, slight opacity; 2, marked opacity; 3, severe opacity; opaque opacity)^30^. Each of the test substances was tested in triplicate using three different bovine eyes on the same day. Monocaprin was tested at 10 times the concentration proposed for the eye drop formulation, demonstrating no irritation even at increased dosage. Povidone iodine was prepared in distilled water at 1%, 2.5%, and 5%, the latter two being concentrations cited in the literature^7–8,11^. A negative control of HPMC and positive control of 0.5 M sodium hydroxide were conducted.

## Data Availability

The datasets generated during and/or analysed during the current study are available from the corresponding author on L.Snyder@kingston.ac.uk.
